# Analysis of reasons and risk factors for non-completion of radiotherapy courses in patients with tumors

**DOI:** 10.3389/fonc.2025.1582201

**Published:** 2025-08-21

**Authors:** Meihua Chen, Kexin Li, Xuan Gao, Xia Ye, Yongjian Ju

**Affiliations:** ^1^ Department of Medical Insurance Management, Nantong First People’s Hospital (Affiliated Hospital 2 of Nantong University), Nantong, Jiangsu, China; ^2^ Department of Radiotherapy, Nantong First People’s Hospital (Affiliated Hospital 2 of Nantong University), Nantong, Jiangsu, China

**Keywords:** tumor, intensity-modulated radiotherapy (IMRT), non-completion of radiotherapy courses (NCRC), risk factors, tumor clinical stage, therapeutic intent

## Abstract

**Purpose:**

Non-completion of radiotherapy courses (NCRC) may happen in patients undergoing intensity-modulated radiotherapy (IMRT). Analyzing the reasons and risk factors for NCRC can lead to possible targeted measures to reduce the incidence rate. This six-year retrospective study will present a cohort analysis of NCRC from a single center.

**Methods:**

Data from patients treated with IMRT between January 2018 and December 2023 were collected for analysis. The collected characteristics included age, gender, residential area, body mass index (BMI), tumor clinical stage, tumor type, tumor location, therapeutic intent, number of fractions, insurance status, treatment completion status, and reasons for NCRC. The radiotherapy process rigorously followed Chinese basic guidelines of quality control for radiotherapy. NCRC was defined as the failure to complete the course after starting the radiotherapy. Patients were categorized into the completion group (CG) and the non-completion group (NCG). Comprehensive analyses included six-year overall NCRC rate and annual trends, inter-group (CG vs. NCG) characteristic disparities, the reasons and risk factors for NCRC.

**Results:**

Among total collected 2,112 IMRT patients, the overall rate of NCRC was 5.68% (n=120), with annual values ranging 4.15–6.69% (p=0.718). The patient-driven reasons for NCRC encompassed: perceiving the final several fractions as non-essential (n=8, 6.67%); perceived insufficient therapeutic efficacy (n=34, 28.33%); financial constraints (n=3, 2.5%). The clinician-driven reasons for NCRC included: severe radiation-induced toxicities (n=39, 32.5%); disease progression (n=30, 25%); death(n=6, 5%). Significant inter-group (CG vs. NCG) differences (p<0.05) were observed in age, gender, body mass index (BMI), therapeutic intent, tumor clinical stage, tumor location, and number of fractions; however significant differences were not observed in the residential area and insurance status. Multivariate analysis revealed that the age, BMI, therapeutic intent, and tumor clinical stage were independent risk factors for NCRC (p<0.05).

**Conclusion:**

Rigorous adherence to the basic guidelines of radiotherapy quality control helped maintain a stable NCRC rate (ranging 4.15%–6.69%). While 62.5% of NCRC cases were attributable to unpredictable radiation-induced toxicities, disease progression or death, the remaining 37.5% (35% due to insufficient awareness of radiotherapy and 2.5% to financial constraints) were potentially preventable. These findings underscore the need for implementing risk-stratified interventions to address modifiable barriers and reduce NCRC rates, particularly in high-risk subgroups characterized by advanced age, lower BMI, later clinical tumor stage, and palliative radiotherapy intent.

## 1 Introduction

While radiotherapy may damage normal tissues during tumor eradication, it remains one of the main means of comprehensive tumor management due to its well-documented benefits in survival outcomes and quality of life metrics (1–3). The therapeutic efficacy of radiotherapy critically depends on delivering the prescribed radiation dose within the scheduled treatment time (4, 5). However, radiotherapy typically involves prolonged treatment courses with multiple fractions, making patients susceptible to treatment interruption or termination due to various influencing factors, resulting to diminish locoregional control, elevate the incidence rate of distant metastasis, and adversely affect overall survival rates (6–11). Consequently, identifying the causes and risk factors for non-completion of radiotherapy courses (NCRC) carries critical implications for developing precision interventions to mitigate its occurrence. This retrospective study conducts a cohort analysis of NCRC using six-year data from patients undergoing intensity-modulated radiotherapy (IMRT) at a tertiary radiotherapy center.

## 2 Materials and methods

### 2.1 Patient data

With approval by the ethics committee of Nantong First People’s Hospital (Affiliated Hospital 2 of Nantong University, Nantong, China), the data from patients who received radiotherapy between January 2018 and December 2023 were systematically extracted from the hospital’s electronic health records. Patients treated with IMRT were included in cohort analysis, while those undergoing two-dimensional (2D) radiotherapy or receiving treatment for benign diseases were excluded. The collected patient characteristics encompassed age, gender, residential area, body mass index (BMI), tumor type, National Comprehensive Cancer Network (NCCN)-classified tumor clinical stage, tumor location, therapeutic intent, insurance status, treatment completion status, and reasons for NCRC.

### 2.2 Radiotherapy process

Our departmental radiotherapy processes were conducted in strict compliance with the Basic Guidelines of Quality Control for Radiotherapy (2017 version) established by the China’s National Cancer Diagnosis and Treatment Quality Control Center, thereby guaranteeing treatment precision and patient safety. A brief description is as follows:

#### 2.2.1 Admission of radiotherapy patient

Patient recruitment strictly followed the Clinical Practice Guidelines for Cancer Diagnosis and Treatment issued by the Chinese Medical Association. For each candidate, board-certified radiotherapy physicians determined the clinical and/or pathological diagnosis, clinical stage, therapeutic intent, and performed a comprehensive pre-treatment assessment to confirm radiotherapy eligibility. For patients with complex clinical presentations, multidisciplinary team (MDT) review was implemented.

#### 2.2.2 Treatment plan design and implementation

Following regular radiotherapy plan design protocols, physicians delineated target volumes and organs at risk based on simulation images using the treatment planning system (Eclipse 8.6; Varian Corp., USA), established prescribed doses and organ-specific constraints; subsequently, physicists designed the IMRT plan. The majority of patients received conventional fractionated radiotherapy with 2 Gy per fraction, the others underwent regimens of 1.8–3 Gy per fraction. The final treatment plan required joint validation by physicians and physicists. During radiotherapy, the medical team conducted weekly evaluations of patients’ conditions and implemented prompt interventions for treatment-related adverse events or disease progression.

NCRC was defined as the failure to complete the course after starting the radiotherapy. In cases of NCRC, the medical team systematically documented the reasons through standardized evaluations; subsequently, these were categorized as two categories, namely patient-driven reasons (i.e., voluntary withdrawal despite medical advice) and clinician-driven reasons (i.e., clinician recommended termination due to severe radiation-induced toxicities or disease progression).

#### 2.2.3 Patient education and informed consent

Prior to commencing radiotherapy, comprehensive patient education was systematically delivered to all individuals, encompassing detailed explanations of radiotherapy mechanisms; treatment schedules (eg., prescription dose, fractionation patterns and total duration, etc.); mandatory attendance requirements; anticipated treatment costs; expected clinical outcomes and potential acute/chronic toxicities, et al. And written informed consent was obtained from every patient after full disclosure of therapeutic parameters and risk-benefit profiles.

### 2.3 Patient grouping and statistical analysis

Patients were categorized into the Completion Group (CG) and Non-Completion Group (NCG). Comprehensive analyses included: six-year overall NCRC rate and annual trends, inter-group(CG vs. NCG) characteristic disparities, the reasons and risk factors for NCRC. IBM SPSS 21.0 (IBM Corp., NY, USA) was used for statistical analysis. Categorical variables (gender, therapeutic intent, etc.) were expressed as the number of cases and percentages(n and %) and analyzed with chi-squared tests (χ² tests); continuous variables (age, BMI) were expressed as mean ± SD and analyzed with unpaired t-tests. The p-values <0.05 indicate statistically significant differences. Variables with p<0.05 underwent multivariable logistic regression to identify independent NCRC risk factors.

## 3 Results

### 3.1 Study cohort and NCRC incidence

Over the six-year study period (2018–2023), 84 patients were excluded from the analysis, comprising 46 patients treated with 2D radiotherapy and 38 patients who underwent radiotherapy for keloid management, with all excluded patients having completed their prescribed treatment courses.

A total of 2112 patients (1,049 males and 1,063 females) who received IMRT were included in the analysis cohort, with a median age of 64 years (range: 17–102 years) and a prescribed dose of 30-70Gy/10–35 fractions within 2–7 weeks. Among these, 120 patients didn’t complete their radiotherapy courses, the overall rate of NCRC was 5.68%, with annual values ranging 4.15–6.69% (p=0.718, p>0.05) (**Table 1**). And the distribution of tumor types and corresponding clinical stages within the NCG (n=120) of our study is shown in **Table 2**.

**Table 1:** Patients in the CG and NCG from 2018 to 2023.

Year	CG (n/%)	NCG (n/%)
2018	284 (94.04)	18 (5.96)
2019	314 (93.45)	22 (6.55)
2020	323 (95.85)	14 (4.15)
2021	307 (93.31)	22 (6.69)
2022	355 (94.16)	22 (5.84)
2023	409 (94.77)	22 (5.23)

Interannual Chi-square test results: χ^2^ = 2.886, p=0.718.

CG, completion group; NCG, non-completion group.

**Table 2:** Tumor type and stage distribution in the NCG.

Tumor type	Cases in the NCG (n)	Proportion compared to cases in the CG+NCG	Cases with different clinical stage (n)			
			I	II	III	IV
Esophageal cancer	29	9.42%		3	12	14
Brain metastasis	21	13.64%				21
Bone metastasis	14	6.87%				14
Pharyngeal cancer	11	8.94%			1	10
Rectal cancer	9	6.16%	1		2	6
Lung cancer	7	4.22%			4	3
Breast cancer	6	1.72%			3	3
Liver cancer	3	14.29%				3
Cervical cancer	3	1.5%				3
Bladder cancer	3	18.75%				3
Prostate cancer	2	1.92%				2
Multiple myeloma	2	22.22%				2
Others	10	3.19%				10
Total	120	–	1	3	22	94

Those with more than two cases were listed separately, and those with only one case each were combined into “Others”.

CG, completion group; NCG, non-completion group.

### 3.2 Reasons for NCRC

The patient-driven reasons encompassed: perceiving the final several sessions as clinically non-essential (n=8, 6.67%); perceived insufficient therapeutic efficacy (n=34, 28.33%); financial constraints (n=3, 2.5%). And the clinician-driven reasons included: severe radiation-induced toxicities (n=39, 32.5%); disease progression (n=30, 25.0%) and death (n=6, 5%) (**Table 3**).

**Table 3:** Reasons for NCRC.

Reasons for NCRC	Cases (n)	n/120 (%)
Patient-driven reasons		
Perceiving the final several fractions as non-essential	8	6.67
Perceived insufficient therapeutic efficacy	34	28.33
Financial constraints	3	2.5
Clinician-driven reasons		
Severe radiation-induced toxicities	39	32.5
Disease progression	30	25
Death	6	5
Total	120	100

NCG, non-completion group; NCRC, non-completion of radiotherapy courses.

### 3.3 Patient characteristics

Baseline characteristics of 2,112 patients are presented in **Table 4**. Comparative analysis revealed the following NCG vs. CG differences: older mean age (+6.3 years: 69.13 ± 10.55 vs. 62.83 ± 12.53); lower mean BMI (-0.91 kg/m²: 22.35 ± 3.10 vs. 23.26 ± 3.87); higher male proportion (+18.9%: 67.5% vs. 48.6%); higher proportion of urban residency (+4.3%: 50.8% vs. 46.5%); higher palliative radiotherapy proportion (+22.1%: 49.2% vs. 27.1%); higher advanced-stage (III-IV) disease prevalence (+39.5%: 96.7% vs. 57.2%); higher proportion of head/neck/chest tumor (+6.6%: 70.8% vs. 64.2%); higher proportion of patients with fractions>25(+13.4%: 51.7% vs. 38.3%); slightly lower uninsured population (-0.7%: 1.7% vs. 2.4%).

**Table 4:** Characteristics of patients in the CG and NCG.

Variables	CG	NCG	*t* or χ^2^	p-value
Age (years)	62.83 ± 12.53	69.13 ± 10.55	−6.284	0.000
Body mass index (kg/m²)	23.26 ± 3.87	22.35 ± 3.10	2.534	0.011
Gender				
Male	968	81	16.182	0.000
Female	1024	39		
Residential area				
Urban	926	61	0.859	0.354
Rural	1066	59		
Therapeutic intent				
Curative-intent radiotherapy	487	38	42.699	0.000
Adjuvant/neoadjuvant radiotherapy	966	23		
Palliative radiotherapy	539	59		
Tumor clinical stage				
Stage I	386	1	158.323	0.000
Stage II	467	3		
Stage III	625	22		
Stage IV	514	94		
Tumor location				
Head and neck	386	41	17.243	0.002
Chest	893	44		
Abdomen	183	13		
Pelvis	504	21		
Extremities	26	1		
Number of fractions				
10–15	143	8	9.708	0.046
16–20	140	9		
21–25	947	41		
26–30	554	46		
31–35	208	16		
Insurance status				
Have	1945	118	0.240	0.624
Do not have	47	2		

Data are presented as numbers or the mean ± standard deviation, unless otherwise indicated.

CG, completion group; NCG, non-completion group.

Significant inter-group (CG vs. NCG) differences (p<0.05) were observed in age, gender, BMI, therapeutic intent, tumor clinical stage, tumor location, and number of fractions; however, significant differences were not observed in the residential area and insurance status.

### 3.4 Risk factors for NCRC

Multivariate analysis revealed that the age, BMI, therapeutic intent, and tumor clinical stage were independent risk factors for NCRC (p<0.05) (**Table 5**).

**Table 5:** Logistic regression analysis of risk factors for NCRC.

Risk factors	B	Exp (B)	95% CI	p-value
Age	0.039	1.040	1.020–1.060	0.000
Body mass index	-0.075	0.928	0.872–0.987	0.018
Gender	0.334	1.396	0.895-2.178	0.141
Therapeutic intent			–	0.000
Curative-intent radiotherapy	-0.931	0.394	0.213–0.730	0.003
Adjuvant/neoadjuvant radiotherapy	-1.371	0.254	0.139-0.462	0.000
Palliative radiotherapy			1	–
Tumor clinical stage			–	0.000
Stage I	-4.311	0.013	0.002–0.097	0.000
Stage II	-3.417	0.033	0.010–0.105	0.000
Stage III	-1.519	0.219	0.134–0.359	0.000
Stage IV			1	–
Tumor location			–	0.575
Head and neck	-0.227	0.797	0.093–6.850	0.836
Chest	-0.529	0.589	0.069–5.032	0.629
Abdomen	-0.709	0.492	0.054–4.463	0.528
Pelvis	-0.625	0.535	0.061–4.693	0.573
Extremities			1	–
Number of fractions			–	0.208
10–15	-0.937	0.392	0.133-1.157	0.090
16–20	-0.667	0.513	0.178-1.479	0.217
21–25	-0.014	0.986	0.437-2.225	0.972
26–30	-0.076	0.927	0.468-1.834	0.827
31–35			1	–

CI, confidence interval.

## 4 Discussion

While the optimal radiotherapy quality assurance processes remain unclear, non-adherence to protocols has been demonstrated to negatively impact clinical outcomes (12). Our six-year departmental data analysis revealed a NCRC rate of 5.68%, which remained stable throughout the observation period and nearly aligned with the 4.6% reported by Nosrati JD et al. (13) and the 4% documented by Arenas M et al. (14). Discrepancies across studies are possibly attributable to regional variations in radiotherapy implementation and differences in patient case mix (15, 16).This suggests that strict adherence to radiotherapy quality control guidelines helps maintain NCRC rates within a stable range. Notably, Kaur J et al. (17) reported a 12% NCRC rate (approximately double our findings), potentially attributable to their cohort comprising 78% of patients receiving 2D radiotherapy versus 100% undergoing IMRT in our cohort. This disparity suggests that emerging radiotherapy technologies integrated with rigorous quality control measures may enhance treatment precision, mitigate adverse effects, and consequently improve treatment adherence–ultimately reducing NCRC rates (18–20).

Among our identified reasons for NCRC, treatment discontinuations driven by clinician determination because of clinically significant radiation-induced toxicities, confirmed disease progression and patient death accounted for 62.5% (75/120) of cases. Although such events remain clinically inevitable, perhaps their incidence can be attenuated through enhanced quality assurance protocols, advanced radiotherapy delivery systems and hypofractionated regimens requiring fewer fractions. Of particular interest was our observation that 37.5% (45/120) of treatment discontinuations were attributable to patient-driven decisions. Within this subgroup, 35% discontinued due to insufficient clinical awareness of radiotherapy, while 2.5% cited financial constraints. This aligns with 35.2% patient-driven discontinuation rate reported by Nosrati JD et al (13). These findings suggest that standard pre-treatment education and informed consent procedures may inadequately address the knowledge gaps among patients regarding radiotherapy expectations and protocols. Current evidence demonstrates that structured health education programs can reduce patient anxiety, improve adverse event management, and enhance treatment adherence (21–23). Perhaps protocol-based interventions incorporating serial psychological evaluations and real-time treatment efficacy communication may better address these cognitive barriers and improve completion rates.

Palliative radiotherapy provides a rapid and effective means of alleviating local symptoms in many advanced cancers during end-of-life care, improving quality of life without imposing significant treatment burden (24, 25). However, patient death during treatment remains clinically significant, as it may preclude potential therapeutic benefit (26). In our study, only 6 of 2,112 patients (0.28%) died during radiotherapy course—a rate significantly lower than the >1.08% mortality reported by Ning MS et al. (26)(where 222 patients died before completing treatment among 20,534 radiotherapy courses; note some patients received multiple courses). This discrepancy may partially reflect distinct cultural preferences and therapeutic approaches in end-of-life care. Specifically in China, most deaths occur at home due to strong preferences among terminally ill patients to return home for their final moments (27–29), which likely lead to the bias of observed mortality for NCRC patients.

Yarn C et al. (30) identified insurance status as a determinant of treatment completion, with additional studies (31, 32) documenting significant financial burdens associated with cancer therapy. In our cohort of 120 NCRC patients, we identified three cases (2.5%) of radiotherapy discontinuation secondary to financial constraints, two of whom were uninsured. These collective findings underscore the potential value of cost-reduction strategies and charitable funding mechanisms to support treatment continuity in vulnerable populations.

Of the collected patient characteristics, age, BMI, therapeutic intent, and tumor clinical stage were identified as independent risk factors for NCRC, these are all indicators reflecting the patient’s physical condition. Regarding the incidence of NCRC (**Table 4**), the data shows that patients with clinical stage IV disease had a significantly higher rate at 15.5% (94/608), far exceeding the rate of 1.73% (26/1478) in patients with other stages;patients receiving palliative radiotherapy had a rate of 9.87% (59/598), which was higher than the 4.03% (61/1514) rate in patients undergoing curative-intent or adjuvant radiotherapy. The likely reason is that patients with advanced clinical stage or undergoing palliative radiotherapy often have a complex overall health condition after years of anti-cancer treatment, and their baseline physical condition tends to be relatively poor. Existing evidence corroborates these findings: Rim CH et al. (33) reported that 19.3% of elderly patients exhibited treatment intolerance during conventional radiotherapy; Some studies (34–36) revealed that malnutrition and significant weight loss were common causes of radiotherapy interruption; Gautam S et al. (37) documented incomplete treatment courses in 10% of palliative radiotherapy patients. Furthermore, although patient gender, tumor location, and number of fractions differed between the CG and NCG groups, multivariate regression analysis indicated that these were not independent risk factors for NCRC (**Table 5**), also none of these three characteristics is associated with the patient’s baseline physical condition. This indicates that the completion of the radiotherapy course is closely related to the patient’s overall physical condition. Consequently, for high-risk subgroups characterized by advanced age, low BMI, advanced tumor clinical stages, and palliative radiotherapy intent, implementing hypofractionated radiotherapy protocols, enhancing prognostication accuracy, and intensifying nutritional support may mitigate these barriers and reduce NCRC incidence.

Several limitations should be acknowledged. Firstly, being a single-center retrospective analysis constrained by departmental radiotherapy equipment and technical capabilities, our study exclusively focused on IMRT patients, potentially introducing regional bias and limiting generalizability. Secondly, this analysis only included nine patient characteristics and did not provide a detailed assessment of psychosocial determinants (such as treatment-related anxiety and family support status) when classifying the causes, resulting in incomplete identification of reasons and risk factors for NCRC. Finally, while we included patient death as a reason for NCRC, due to the small number of deaths, we did not analyze the timing of death relative to radiotherapy cessation. Future studies shall investigate how applied new radiotherapy technologies, enhanced patient education throughout the radiotherapy process, enhanced radiation-induced toxicity early-warning systems, and optimized multidisciplinary supports impact NCRC rates. Additionally, multicenter collaborative efforts to standardize quality control protocols, refine stratification of NCRC factor to identify modifiable barriers, and develop evidence-based interventions for reducing NCRC rates are worth studying.

## 5 Conclusion

The six-year single-center data demonstrated that rigorous adherence to radiotherapy quality control guidelines helped maintain a stable NCRC rate (ranging 4.15%–6.69%). While 62.5% of NCRC cases were attributable to unpredictable radiation-induced toxicities, disease progression or death, the remaining 37.5% (35% due to insufficient awareness of radiotherapy and 2.5% to financial constraints) were potentially preventable. These findings underscore the need for implementing risk-stratified interventions to reduce NCRC rates, particularly in high-risk subgroups characterized by advanced age, lower BMI, later clinical tumor stage, and palliative radiotherapy intent. The possible targeted clinical interventions, such as using hypofractionated radiotherapy and improving prognostication, implement of psychological evaluations and real-time treatment efficacy communication, strengthening nutritional support, establishing cost reduction strategies and charitable funding mechanisms for vulnerable groups et al., may better address these modifiable barriers and reduce NCRC rates.

## Data Availability

The original contributions presented in the study are included in the article/supplementary material. Further inquiries can be directed to the corresponding author.
